# A Review of Nonoccupational Pathways for Pesticide Exposure in Women Living in Agricultural Areas

**DOI:** 10.1289/ehp.1408273

**Published:** 2015-01-30

**Authors:** Nicole C. Deziel, Melissa C. Friesen, Jane A. Hoppin, Cynthia J. Hines, Kent Thomas, Laura E. Beane Freeman

**Affiliations:** 1 Division of Cancer Epidemiology and Genetics, National Cancer Institute, National Institutes of Health, Department of Health and Human Services, Bethesda, Maryland, USA; 2 Department of Environmental Health Sciences, Yale School of Public Health, Yale University, New Haven, Connecticut, USA; 3 Department of Biological Sciences, North Carolina State University, Raleigh, North Carolina, USA; 4 Division of Surveillance, Hazard Evaluation and Field Studies, National Institute for Occupational Safety and Health, Centers for Disease Control and Prevention, Cincinnati, Ohio, USA; 5 National Exposure Research Laboratory, U.S. Environmental Protection Agency, Research Triangle Park, North Carolina, USA

## Abstract

**Background:**

Women living in agricultural areas may experience high pesticide exposures compared with women in urban or suburban areas because of their proximity to farm activities.

**Objective:**

Our objective was to review the evidence in the published literature for the contribution of nonoccupational pathways of pesticide exposure in women living in North American agricultural areas.

**Methods:**

We evaluated the following nonoccupational exposure pathways: paraoccupational (i.e., take-home or bystander exposure), agricultural drift, residential pesticide use, and dietary ingestion. We also evaluated the role of hygiene factors (e.g., house cleaning, shoe removal).

**Results:**

Among 35 publications identified (published 1995–2013), several reported significant or suggestive (*p* < 0.1) associations between paraoccupational (*n* = 19) and agricultural drift (*n* = 10) pathways and pesticide dust or biomarker levels, and 3 observed that residential use was associated with pesticide concentrations in dust. The 4 studies related to ingestion reported low detection rates of most pesticides in water; additional studies are needed to draw conclusions about the importance of this pathway. Hygiene factors were not consistently linked to exposure among the 18 relevant publications identified.

**Conclusions:**

Evidence supported the importance of paraoccupational, drift, and residential use pathways. Disentangling exposure pathways was difficult because agricultural populations are concurrently exposed to pesticides via multiple pathways. Most evidence was based on measurements of pesticides in residential dust, which are applicable to any household member and are not specific to women. An improved understanding of nonoccupational pesticide exposure pathways in women living in agricultural areas is critical for studying health effects in women and for designing effective exposure-reduction strategies.

**Citation:**

Deziel NC, Friesen MC, Hoppin JA, Hines CJ, Thomas K, Beane Freeman LE. 2015. A review of nonoccupational pathways for pesticide exposure in women living in agricultural areas. Environ Health Perspect 123:515–524; http://dx.doi.org/10.1289/ehp.1408273

## Introduction

Most evidence for health effects of pesticides in adults comes from studies of occupationally exposed men (McDuffie 1994, 2005). Relatively less is known about pesticide-related health effects in women, and there may be sex-specific risk differences with respect to reproductive toxicity and hormonally driven cancers (Caserta et al. 2008; Ward et al. 2010). In addition, comparatively little is known about nonoccupational pesticide exposure pathways. Although these pathways may contribute less than occupational exposures in occupationally exposed individuals, these pathways are expected to be important in nonoccupationally exposed populations, particularly for those living in regions of intense crop production. Some studies have observed that pesticide exposures in women living in agricultural areas are consistent with the high end of the exposure distribution for the general population (Arbuckle and Ritter 2005; Bradman et al. 2005, 2007; Castorina et al. 2010; Curwin et al. 2007). However, the contribution of nonoccupational exposure pathways to pesticide exposure in agricultural women is not well-characterized. Understanding their pesticide exposure pathways is integral to a more comprehensive evaluation of health risks of pesticides among women.

The objective of this review was to identify the important pathways and gaps in the literature through consideration of all published reports of nonoccupational pesticide exposure in women living in agricultural areas in North America. Women in agricultural areas may be exposed to pesticides if they are farmers or farmworkers, live with a farmer or farmworker (i.e., in a “farm home”), or live in a home in an agricultural area without any farmer or farmworker residents (i.e., a “non-farm home”). We excluded the “occupational pathway,” defined here as personal mixing and applying of pesticides on a farm. Nonoccupational pathways include paraoccupational, agricultural drift (primary and secondary), residential pesticide use, and dietary ingestion. We define paraoccupational exposures as those occurring through the introduction of pesticides into the home by household members who use or contact pesticides at work or from bystander exposure during pesticide applications (e.g., a wife engaging in outdoor farm tasks not involving contact with pesticides, such as driving a tractor). We define primary agricultural drift as that which occurs from the transport of pesticides to non-treatment sites at the time of application, whereas secondary drift involves the volatilization and movement of pesticide residues from soil and plants or the movement of pesticide-laden dust or soil by wind after the time of application (Ward et al. 2006). We consider the residential pesticide use exposure pathway to be that which occurs from the application of pesticides to the home, lawn, or garden. Dietary ingestion occurs from drinking water or eating food containing pesticide residues. Exposures experienced via these pathways may be modified by hygienic practices undertaken to reduce pesticide exposures, such as separate laundering of pesticide-contaminated clothing or changing work clothes and shoes prior to entering the home. We reviewed the evidence for the contribution of each exposure pathway individually.

## Methods

We identified relevant pesticide exposure studies published in the English language through September 2013. We searched PubMed (http://www.ncbi.nlm.nih.gov/pubmed/), Scopus (http://www.elsevier.com/scopus), Web of Science (http://www.isiknowledge.com), and Google Scholar (http://scholar.google.com) and checked reference lists of pertinent articles. Most studies were obtained using a PubMed search using the following terms: “environmental exposure[MeSH] AND pesticides[MeSH] AND (home OR household OR indoor).” To be included in this review, publications had to meet the following criteria: *a*) be conducted in North America, *b*) focus on exposure as the outcome measure, *c*) include biological and/or environmental measurements of women living in agricultural areas or environmental measurements from homes in agricultural areas, and *d*) address at least one of the exposure pathways of interest or hygiene factors. We focused on studies conducted in North America because of international variability in agricultural and household practices due to differences in climate, types of pests, types of crops, and pesticide regulations. We used inclusive criteria and report on the findings from all studies with at least five samples. We excluded papers that presented pilot/preliminary data if the data were included in a subsequent, more comprehensive publication. For studies with environmental measures, we focused on pesticide levels in residential dust (including bulk dust and surface wipe samples) and assumed they would be representative of women’s exposures, although they would be applicable to all household members. Dust measurements may represent chronic residential pesticide exposure because chemicals in indoor dust resist degradation due to limited sunlight, microbial activity, and moisture (Lewis et al. 1994; Simcox et al. 1995). Publications reported dust measurements as concentrations (mass of pesticide per mass of dust) and/or loadings (mass of chemical per surface area). We excluded results from air sampling because only three studies included it, and these measurements were predominantly below the detection limit (Curwin et al. 2005; Lu et al. 2004; Weppner et al. 2006). We excluded results from food samples because only one pilot study measured pesticides in food (Melnyk et al. 1997). Studies with biological measures included analysis of pesticides or pesticide metabolites in blood or urine specimens collected from women in agricultural areas.

## Results

Characteristics of the 35 publications (published 1995–2013) meeting our search criteria are presented in Table 1. The majority (29 of 35) were attributed to 10 larger studies or research groups. Fourteen of 35 (40%) publications were conducted in the northwestern United States in Washington or Oregon, 6 (17%) included populations in Iowa, 5 (14%) in California, 5 (14%) in North or South Carolina (SC), 4 (11%) in Canada, and 3 (9%) in Minnesota. These geographic regions reflect differences in crop types and application methods, with orchard farms dominating the studies in the northwestern United States, corn and soybean farms found commonly in the Iowa studies, and varied crops (vineyards, fruits, vegetables) in California. Sample sizes ranged from 6 to 816 participants or households (residences and/or occupants). Some studies focused on women specifically, including those from the Farm Family Exposure Study (Minnesota and South Carolina), the Iowa farm family exposure study, and the Ontario pesticide exposure assessment study (Canada). The Center for Health Assessment of Mothers and Children of Salinas (CHAMACOS) study (California) included > 600 pregnant women, but the target population was the children. Twenty-eight publications included environmental samples from the home (26 dust and 4 water; 2 both). Eleven publications included biological samples, 4 of which also included environmental samples. The publications predominantly covered organophosphate insecticides, such as chlorpyrifos (*n* = 19), azinphos-methyl (*n* = 14), phosmet (*n* = 13), and diazinon (*n* = 12), as well as common agricultural herbicides such as 2,4-dichlorophenoxyacetic acid (2,4-D) (*n* = 9) and atrazine (*n* = 8); only 3 studies included fungicides (see Supplemental Material, Table S1).

**Table 1:** Evidence for nonoccupational pesticide exposure pathways in reviewed literature.

Study, source	Location, time period, population	Crops	Environmental sample^*a *^	Biological sample^*b *^	Paraoccupational	Agricultural drift	Residential use	Ingestion	Statistical analyses
Agricultural Health Pilot Study
Melnyk et al. 1997	IA, NC 1994 6 households	Cattle, grains, soybeans, fruit, vegetables	Water	NA	NA	NA	NA	Descriptive	Summary statistics
California Childhood Leukemia Study/Fresno pesticide exposure study
Gunier et al. 2011	CA 2001–2006 89 households	NA	Dust	NA	+	+	+	NA	Multivariable regression
Deziel et al. 2013	CA 2003–2005 21 households	NA	Dust	NA	NA	+	+	NA	Multivariable regression
CHAMACOS
Bradman et al. 2007	CA 1999–2000 426 pregnant women	Vegetables, vineyards, orchards	NA	Blood	NA	o	NA	NA	Multivariable regression
Harnly et al. 2009	CA 1999–2000 197 households	Vegetables, vineyards, orchards	Dust	NA	+	+	NA	NA	Multivariable regression
Huen et al. 2012	CA 1999–2000 234 mothers	Vegetables, vineyards, orchards	NA	Blood, urine	o	o	NA	NA	NA
Farm Family Exposure Study
Acquavella et al. 2004	MN, SC 2000–2001 48 households	NA	NA	Urine	o	o	NA	NA	Summary statistics
Alexander et al. 2006	MN, SC 2000–2001 34 households	NA	NA	Urine	+	o	NA	NA	Nonparametric tests, multivariable regression
Alexander et al. 2007	MN, SC 2000–2001 34 households	NA	NA	Urine	+	o	NA	NA	Summary statistics, multivariable regression, nonparametric tests
For Healthy Kids study
Coronado et al. 2004	WA 1999 156 households	Orchards, berries, grapes, hops	Dust	NA	+	NA	NA	NA	Chi-square test
Coronado et al. 2006	WA 1999 156 households	Orchards, berries, grapes, hops	Dust	NA	+	NA	NA	NA	Summary statistics
Curl et al. 2002	WA 1999 156 households	Tree fruit, berries, grapes, hops	Dust	NA	+	o	NA	NA	ANOVA, correlations
Coronado et al. 2011	WA 2005–2006 109 households	Orchards, berries, grapes, hops	Dust	Urine	+	o	NA	NA	Summary statistics
Coronado et al. 2012	WA 2005 95 households	Orchards, berries, grapes, hops	Dust	Urine	NA	NA	NA	NA	Chi-square, nonparametic tests
Thompson et al. 2008	WA 1999–2003 210 households	Orchards, berries, grapes, hops	Dust	NA	NA	NA	NA	NA	Summary statistics
Iowa farm family pesticide exposure study
Curwin et al. 2005	IA 2001 50 households	Corn, soybeans	Dust	NA	+	o	o	NA	Mixed effects models
Curwin et al. 2007	IA 2001 50 households	Corn, soybeans	Dust	Urine	+	NA	NA	NA	Mixed effects models
Iowa pesticide exposure studies
Golla et al. 2012	IA 2005 32 households	Corn	Dust	NA	+	o	o	NA	ANOVA
Lozier et al. 2012	IA 2007–2009 30 households	Corn	Dust	NA	+	o	o	NA	ANOVA, single and multivariable regression
Ontario pesticide exposure assessment study
Arbuckle et al. 2006	Ontario, Canada 1996 32 households	Livestock, grains, oilseeds, fruits, vegetables	Dust, water	Urine	o	NA	NA	Descriptive	Nonparametric tests, correlation
Arbuckle and Ritter 2005	Ontario, Canada 1996 126 households	Livestock, grains, oilseeds, fruits, vegetables	NA	Urine	+	NA	NA	NA	Nonparametric tests, correlation
Oregon pesticide exposure studies
McCauley et al. 2001	OR 1997 96 households	Orchards, vegetables, berries	Dust	NA	NA	++/o	o	NA	NA
McCauley et al. 2003	OR 1998 24 households	Orchards	Dust	NA	+	o	o	NA	*t*-Test, nonparametric test, ANOVA, correlations
McCauley et al. 2006	OR NA 10 households	Orchards	Dust	NA	NA	NA	NA	NA	Nonparametric test
University of Washington studies
Fenske et al. 2002	WA 1995 75 households	Orchards	Dust	NA	+	+	NA	NA	Nonparametric tests
Lu et al. 2000	WA 1995 76 households	Orchards	Dust	NA	+	+	o	NA	Nonparametric tests
Lu et al. 2004	WA 1998 6 households	Orchards	Dust, water	NA	NA	NA	NA	Descriptive	Summary statistics
Simcox et al. 1995	WA 1992 59 households	Orchards	Dust	NA	+	+	NA	NA	Nonparametric tests, correlation
Weppner et al. 2006	WA NA 6 households	Potatoes	Dust	NA	NA	o	NA	NA	Summary statistics
Other studies
Freeman et al. 2004	TX 2000–2001 27 households	NA	Dust	NA	NA	NA	+/o	NA	Nonparametric tests
Fitzgerald et al. 2001	Alberta, Canada 1995–1996 816 households	NA	Water	NA	NA	NA	NA	Descriptive	Descriptive
Quandt et al. 2004	VA, NC 2001 41 households	NA	Dust	NA	NA	+	NA	NA	Multivariable regression
Richards et al. 2001	AR NA 11 households	Rice	Dust	NA	NA	Descriptive	NA	NA	Descriptive
Semchuk et al. 2003	Saskatchewan, Canada 1996 154 women	Grain/wheat	NA	Blood	+	NA	NA	NA	Multivariable regression
Ward et al. 2006	IA 1998–2000 112 households	Corn/soybeans	Dust	NA	+	+	NA	NA	Multivariable regression, summary statistics
Abbreviations: +, positive association observed for at least one pesticide (*p* < 0.1); o, no observed associations (*p* > 0.1); ANOVA, analysis of variance; AR, Arkansas; CA, California; IA, Iowa; MN, Minnesota; NA, not available or not applicable; NC, North Carolina; OR, Oregon; SC, South Carolina; TX, Texas; VA, Virginia; WA, Washington. ^***a***^Dust samples include bulk dust and dust wipes. ^***c***^Samples collected from women living in agricultural areas (not men or children).									

Here, we describe the evidence related to each pathway separately by environmental and biological monitoring, but not by individual pesticide because of insufficient information. We determined whether studies observed associations that were suggestive (0.05 < *p* < 0.1), statistically significant (*p* < 0.05), null (*p* > 0.1), or descriptive (no comparison groups or no *p*-values or confidence intervals provided). In some studies, relationships differed by pesticide or exposure metric. Overall, we classified studies that reported at least one suggestive or statistically significant association as providing evidence for a particular pathway in Table 1, with the details described and discussed in the text.

### Paraoccupational Exposure

Twenty-two publications investigated the paraoccupational exposure pathway, 13 with residential dust samples only, 6 with biological samples only, and 3 with both (Table 1).

*Residential dust.* Nine publications evaluated the paraoccupational pathway by comparing pesticide concentrations in residential dust in farm homes to non-farm homes, and two studies compared pesticide concentrations in residential dust in farm homes during planting and non-planting seasons. Because most farm homes were also located near treated fields, it was difficult to disentangle the paraoccupational and agricultural drift pathways. Several publications accounted for drift by adjusting for proximity to farmland in multivariable regression models or by restricting the analysis to all homes beyond a specified distance from treated fields. After adjustment for nearby agricultural applications, Gunier et al. (2011) observed that farm homes had higher levels of chlorpyrifos and simazine (but not five other pesticides evaluated) in residential dust compared with non-farm homes. In a University of Washington study (Simcox et al. 1995), dust levels of azinphos-methyl, chlorpyrifos, parathion, and phosmet were 3–15 times higher in farm homes compared with non-farm homes. The authors observed interactions for some pesticides between farm and non-farm homes and proximity to treated crops, making it difficult to assess the independent contribution of each of these factors. In another University of Washington study, levels of chlorpyrifos, azinphos-methyl, and phosmet, commonly used insecticides in the region, were significantly higher in farm homes compared with non-farm homes, all located > 400 m from treated fields (Fenske et al. 2002; Lu et al. 2000). These authors found no association for parathion, the use of which had been discontinued in the area prior to the study, suggesting that the observed differences were due to more recent usage. In CHAMACOS (Harnly et al. 2009), farm homes compared with non-farm homes had higher dust levels of iprodione—but not chlorpyrifos, *trans*-permethrin, diazinon, or dacthal—after adjustment for agricultural pesticide use near the home; 15 other pesticides were not evaluated due to detection rates < 5%.

Three Iowa studies found that adjustment for drift had no impact on the paraoccupational relationships. In the Iowa farm family pesticide exposure study (Curwin et al. 2005), concentrations of atrazine, metolachlor, chlorpyrifos, acetochlor, alachlor, glyphosate, and 2,4-D were higher in farm homes than in non-farm homes, but the differences were significant only for atrazine and metolachlor. Homes of farmers who had applied atrazine, metolachlor, chlorpyrifos, and glyphosate within 7 days before sampling had higher levels of the respective chemicals in dust compared with non-farm homes and farm homes that did not apply the chemical (Curwin et al. 2005). Golla et al. (2012) found that atrazine concentrations in residential dust in Iowa farm homes were an order of magnitude higher during the planting season, when atrazine is widely applied, than during the non-planting season. Lozier et al. (2012) also observed higher atrazine loadings in the application season compared with the off-season in same-room samples in Iowa homes of agricultural commercial pesticide applicators.

Four publications that did not present comparisons specifically accounting for drift reported higher detection rates and concentrations of at least one pesticide in residential dust in farm homes compared with non-farm homes. Ward et al. (2006) found that both detection rates and concentrations of six agricultural herbicides in residential dust were approximately four times higher in Iowa farm homes compared with non-farm homes. In a study of organophosphates commonly used in agriculture in Washington State (Lu et al. 2000), diazinon and azinphos-methyl were more frequently detected in farm homes than in non-farm homes, but chlorpyrifos and phosmet were not quantifiable in any homes. In the For Healthy Kids study, residential dust concentrations of azinphos-methyl and phosmet were significantly five times and three times higher in Washington State farm homes than non-farm homes, respectively (Coronado et al. 2011); both pesticides were commonly used during the study period. Within the farm homes in the For Healthy Kids study, researchers observed a significant correlation (*r* = 0.52) between azinphos-methyl concentrations in house dust and dust from commuters’ vehicles, providing additional support for the paraoccupational pathway (Coronado et al. 2006; Curl et al. 2002). Results for other organophosphates measured in the study (malathion, diazinon, methyl parathion, and chlorpyrifos) were not reported due to low detection rates or limited use in the study region.

Six publications evaluated the impact of specific tasks on the paraoccupational exposure pathway. In the For Healthy Kids study, farmworkers who reported working with pome fruit (e.g., apples, pears) versus non-pome fruit (Coronado et al. 2006), thinning orchards versus no thinning (Coronado et al. 2004), or pruning versus no pruning (Coronado et al. 2004) had significantly higher levels or greater percent detection of azinphos-methyl in the dust in their homes and/or the vehicles they used to commute. No associations with residential or commuter vehicle dust levels were observed for farmworkers who reported mixing/loading/applying pesticides; harvesting or picking; loading plants, fruits, or vegetables; sorting plants, fruits, or vegetables; planting or transplanting; or irrigating (Coronado et al. 2004). McCauley et al. (2003) reported that median levels of the summed concentrations of four organophosphate pesticides (azinphos-methyl, chlorpyrifos, malathion, and phosmet) were higher in Oregon farmworker homes if at least one person was involved in tree thinning, compared with homes with no one reporting that task. In a University of Washington study (Fenske et al. 2002), median residential dust concentrations and loadings of chlorpyrifos and parathion were statistically significantly higher in homes of farmers who applied pesticides versus those who did not. In an Iowa study, Golla et al. (2012) observed that atrazine concentrations were higher in homes of farmers who personally mixed and applied atrazine compared with those of farmers who did not. In another study in Iowa, Lozier et al. (2012) did not observe differences in atrazine loadings among homes of applicators, mixers, or applicator/mixers in samples collected during peak application season.

*Pesticide biomarkers.* Six publications reported results from urinary pesticide biomarker measurements in women the day before, the day of, and ≥ 1 day after their husbands applied the pesticides of interest. Comparisons of pre- and postapplication biomarker levels in these women did not suggest increased exposure as a result of a specific pesticide application event. In the Iowa farm family exposure study (Curwin et al. 2007), the estimated geometric mean concentration of the urinary metabolite of metolachlor was 4 times higher (not statistically significant) over the sampling period in women whose husbands applied the chemical compared with those whose husbands did not. No differences were observed for urinary biomarkers of atrazine, chlorpyrifos, or glyphosate. However, the correlations between urinary pesticide concentrations between husband and wife across the pre-, during-, and postapplication periods combined were moderate to high for metolachlor (0.66), atrazine (0.43), chlorpyrifos (0.61), and glyphosate (0.59) (Curwin et al. 2007). In the same study, the urinary biomarkers of atrazine and chlorpyrifos in women in farm homes during the application event were significantly or suggestively higher than the levels in women in non-farm homes; no differences were observed for biomarkers of metolachlor or glyphosate (Curwin et al. 2007). In the Ontario pesticide exposure assessment study (Arbuckle and Ritter 2005; Arbuckle et al. 2006), in which husbands applied at least one of the two herbicides 2-methyl-4-chlorophenoxyacetic acid (MCPA) and 2,4-D, the percentage of detectable urinary biomarkers of these chemicals did not differ in women in the days before, during, and after their husbands applied the respective chemical(s). The percentage of nondetects among the women was approximately 80% throughout; in contrast, the husbands’ urinary biomarker concentrations were approximately four times higher after applying the chemicals. In the same study, Arbuckle et al. (2006) observed no correlation in urinary 2,4-D concentrations between wives and husbands at the time of application. Higher correlations between spouses and applicators were observed for several additional herbicides, including dichlorprop (*r* = 0.57), mecoprop (0.52), and 4-(4-chloro-2-methylphenoxy)butyric acid (*r* = 0.70), although it was not clear whether any were applied at the time of sampling (Arbuckle and Ritter 2005). In the Farm Family Exposure Study, there were negligible changes in urinary biomarker concentrations of glyphosate, 2,4-D, and chlorpyrifos on the day of application or 3 days following application in spouses whose husbands applied the chemical, even when the applicator’s exposure increased during that same time period (Acquavella et al. 2004; Alexander et al. 2006, 2007). Correlations between applicator and spouse biomarker levels were not reported in these publications.

There was modest evidence that spouses who were “present” while their husbands applied the pesticide(s) had higher urinary pesticide levels, although it was not clear how “presence” was defined. For example, in the Farm Family Exposure Study, spouses who were present at some time while their husbands applied 2,4-D (Alexander et al. 2007) or chlorpyrifos (Alexander et al. 2006), as documented by a trained observer, had urinary concentrations of the respective pesticide biomarkers approximately 1.5 times those of women who were not present at any time during the application; differences were not statistically significant. The percent detection (2–4%) of glyphosate in spouses whose husbands had applied it was too low to evaluate the impact of spouse presence during application (Acquavella et al. 2004). In the Ontario Pesticide Exposure Assessment study, median urinary levels of 2,4-D, but not MCPA, of women who were “outside” while their husband applied the specific herbicide were statistically significantly higher compared with all other women (Arbuckle and Ritter 2005).

Two publications compared pesticide biomarkers in women living in farm homes with those in women living in non-farm homes, independent of a specific application event. Huen et al. (2012) observed no differences in the percentage of detectable blood organophosphate levels. The odds of detection of bromoxynil phenol in the plasma of women living with a grain farmer were elevated (not statistically significantly) compared with women who did not live with a grain farmer (Semchuk et al. 2003).

### Agricultural Drift

Twenty-two publications addressed agricultural drift, 16 using residential dust only, 5 with biomarkers only, and 1 with both (Table 1).

*Residential dust.* Six publications reported associations between concentrations of pesticides in residential dust and proximity to treated farmland, a commonly used surrogate for drift. A University of Washington study observed significantly higher levels of azinphos-methyl, chlorpyrifos, and parathion in residential dust from farm and non-farm homes within 50 ft of treated land compared with homes located farther away (Simcox et al. 1995). When restricted to farm homes only, levels of azinphos-methyl and parathion (but not chlorpyrifos) remained significantly elevated within 50 ft of treated land (Simcox et al. 1995). In another University of Washington study, concentrations of azinphos-methyl (Lu et al. 2000) and chlorpyrifos (Fenske et al. 2002) were significantly higher in farm homes within 200 ft of treated orchards compared with homes farther away. No such associations were observed for phosmet (Lu et al. 2000), which was also commonly used, or parathion (Fenske et al. 2002), which was historically but not currently used. McCauley et al. (2001) reported that concentrations of azinphos-methyl in farm homes decreased significantly by 18% when the distance from agricultural fields doubled. Quandt et al. (2004) observed that surface wipes inside farm homes in Virginia and North Carolina yielded higher odds of detection, but not odds of higher concentrations, of at least one of six agricultural-use pesticides (disulfoton, esfenvalerate, lindane, oxyfluorfen, pendimethalin, simazine) in homes that were “within a short walk” of farmland. Richards et al. (2001) reported that three of eight homes within 125 m of a treated rice field had detectable levels of the commonly used propanil, while none of the homes located further away did.

Seven publications did not observe an association between distance to agricultural land and pesticide exposures. In two study populations of farm homes in the For Healthy Kids study, proximity to farmland was not associated with increased residential dust concentrations of azinphos-methyl (Coronado et al. 2011; Curl et al. 2002) or phosmet (Coronado et al. 2011). In the Iowa farm family pesticide exposure study, Curwin et al. (2005) found no association between distance to treated farmland and concentrations of atrazine in residential dust in non-farm homes. Two studies of Iowa farm homes (Golla et al. 2012; Lozier et al. 2012) reported no relationship between distance from home to crop fields and atrazine levels in dust. Similarly, in a study of Oregon farm homes, no association was seen between total organophosphate levels and distance to the nearest active orchard (McCauley et al. 2003). Weppner et al. (2006) studied six homes in central Washington State located within 200 m of potato fields and found no increase in indoor methamidophos surface residues following aerial applications of methamidophos to the fields.

Four studies incorporated additional information to the distance metrics, such as crop acreage, amount of pesticide applied, and wind direction, to assess the relationship between drift and levels of pesticides in the dust. Ward et al. (2006) found that the frequency of detection of at least one of six agricultural herbicides studied (acetochlor, alachlor, atrazine, benazon, fluazifop-*p*-butyl, and metolachlor) increased 6% with each 10-acre increase in crop acreage up to the maximum buffer radius of 750 m, even after adjusting for the presence of a farmworker resident. In addition, for each 10-acre increase in crops within 750 m, there was a 1.05-fold increase in agricultural herbicide concentration. There was no clear pattern when using the simpler metric of distance to treated land. Gunier et al. (2011) observed that concentrations of five (chlorpyrifos, dacthal, iprodione, simazine, and phosmet) of seven (not carbaryl or diazinon) pesticides that were applied agriculturally within 1,250 m of a home during the prior year were present at higher concentrations in the residential dust compared with homes without application of the respective pesticide. Associations remained after adjusting for the presence of farmworkers. In CHAMACOS (Harnly et al. 2009), each kilogram per day increase in application of chlorpyrifos, dacthal, and iprodione near the home (up to a 9-mi^2^ area or ~ 2,800-m radius) was associated with increased pesticide dust concentrations after adjustment for farmworker residents. Conversely, no relationship was seen for permethrin or diazinon. Harnly et al. (2009) observed no increase in residential dust loadings or concentrations when using a simpler distance metric (i.e., comparing homes within 60 m of a field to those located farther away). In the Fresno pesticide exposure study, application of trifluralin (but not eight other pesticides evaluated) within a 1,250-m buffer around a home was significantly associated with concentrations in the dust after adjusting for other factors, such as residential pesticide use (Deziel et al. 2013).

*Pesticide biomarkers.* Five publications examining the influence of agricultural drift on pesticide biomarker levels in women observed no associations. In the For Healthy Kids study, Coronado et al. (2011) observed a 23% reduction in the nonspecific organophosphate urinary metabolite dimethylthiophosphate with each mile from farmland in non-farmworkers who were 81% women, but the relationship was not suggestive or statistically significant (95% confidence interval: –45, 11%). In the Farm Family Exposure Study, proximity to treated farmland was not associated with increased urinary 2,4-D (Alexander et al. 2007), 3,5,6-trichloro-2-pyridinol (a chlorpyrifos metabolite) (Alexander et al. 2006), or glyphosate (Acquavella et al. 2004). In CHAMACOS, living within 200 ft of a field was not associated with higher detection of blood levels of organophosphates (Huen et al. 2012). Also in CHAMACOS, Bradman et al. (2007) observed no association between living within 60 m of an agricultural field and higher serum levels of the organochlorines dichlorodiphenyltrichloroethane (DDT), dichlorodiphenyldichloroethene (DDE), hexachlorocyclohexane (HCH), and hexachlorobenzene (HCB); however, these pesticides had been banned or restricted in the United States prior to the study period, and serum levels were mainly related to birth in Mexico, where the organochlorines have had more recent use.

### Residential Pesticide Use

Nine publications examined the relationship between personal or professional pesticide applications in homes and residential dust measurements of pesticides in farm homes or homes in agricultural areas (Table 1). No biomonitoring studies evaluated this pathway.

Three publications reported associations between pesticide applications in homes in agricultural areas and residential dust measurements for at least one pesticide. These studies asked specific questions about pest treatment practices and focused on homes located in agricultural areas, not specifically farm homes. In the California Childhood Leukemia Study (Gunier et al. 2011), households reporting treatments for fleas/ticks or outdoor professional pest treatments (compared with households not receiving treatment) had significantly higher concentrations of chlorpyrifos and diazinon, respectively, in carpet dust after adjustment for density of agricultural pesticide use within a 1,250-m buffer around the home. No relationships between pest treatments and carpet dust concentrations were seen for carbaryl, dacthal, phosmet, simazine, or iprodione, although residential uses for iprodione were phased out in 1998. In the Fresno pesticide exposure study (Deziel et al. 2013), analyses adjusted for agricultural pesticide applications showed that treatment for bees/wasps/hornets was associated with significantly higher concentrations of chlorpyrifos and that treatment for lawn/garden pests was associated with higher concentrations of diazinon and piperonyl butoxide. Treatment for ants/flies/roaches was associated with significantly higher concentrations of carbaryl, but lower concentrations of piperonyl butoxide and simazine. Homes with professional outdoor treatments (vs.those with no professional treatments) had significantly higher concentrations of permethrin, cypermethrin, cyfluthrin, and diazinon. In an agricultural area of the mid-Rio Grande Valley in Texas on the U.S./Mexico border, loadings of demeton-O were marginally correlated (Spearman *r* = 0.24, *p* = 0.08) with the number of locations within a home where pesticides were applied (Freeman et al. 2004); no significant or suggestive correlations with use were observed for demeton-S, fonofos, diazinon, disulfoton, methyl parathion, fenitrothion, or malathion. Because of reports of potential misuse of agricultural pesticides in the community, the investigators included pesticides with and without approval for residential use. Associations between residential pesticide use and dust levels of pesticides would be expected only if the pesticides’ active ingredients were present in the products used; however, the studies did not generally collect that information.

Six publications reported null or mostly null associations between residential pesticide use and pesticide concentrations in dust. These studies included predominantly farm homes, did not always account for agricultural use, and included some pesticides not permitted or commonly used residentially. McCauley et al. (2001) observed no association between family use of pest control products and levels of azinphos-methyl in farm homes; azinphos-methyl is not registered for residential use. In another study, McCauley et al. (2003) found no association between pesticide use in homes—compared with no use—and levels of total organophosphate residues. Lu et al. (2000) observed no association between residential use of organophosphates in homes, including uses specifically on pets or lawns and gardens and levels of pesticides in residential dust. In farm and non-farm homes in the Iowa farm family pesticide exposure study (Curwin et al. 2005), none of the three self-reported residential use variables (use of an insecticide, treating a lawn with pesticides, and spraying a garden with pesticides) were associated with concentrations of chlorpyrifos, glyphosate, and 2,4-D—pesticides with both agricultural and residential uses. Also in that study, self-reported use of an insecticide was associated with atrazine concentrations in the residential dust of farm homes; however, atrazine is not an insecticide and is not commonly used residentially in Iowa U.S. Environmental Protection Agency (EPA) 2006], so the reason for this association is unknown. Golla et al. (2012) and Lozier et al. (2012) observed no association of the application of pesticides in the home or to the lawn with dust levels of atrazine.

### Ingestion

Four studies measured pesticides in drinking water in farm homes. In the Ontario pesticide exposure assessment study (Arbuckle et al. 2006), 20% of the 122 farm homes had drinking water with measurable levels of at least 1 pesticide, most commonly atrazine. Only 1% had detectable levels of 2,4-D, and 3% had detectable levels of MCPA, although in each home a farmer had used at least 1 of the 2 chemicals at the time of study. In a study of 816 farm homes using well water in Alberta, Canada (Fitzgerald et al. 2001), 3% of homes had measurable levels of at least 1 of 8 herbicides in their tap water, with 2% positive for MCPA and 1% positive for 2,4-D. The 6 other herbicides (dicamba, bromoxynil, fenozaprop, diclofop-methyl, trifluralin, and triallate) were not detected. In a study of 6 farm homes in Washington State, none of the commonly used organophosphate pesticides analyzed (azinphos-methyl, chlorpyrifos, diazinon, dichlorovos, or phosmet) were detected in drinking water (Lu et al. 2004). Similarly, in a pilot study within the Agricultural Health Study (Melnyk et al. 1997), none of the 30 target pesticides analyzed were detected in drinking water samples from 6 farm homes.

### Hygiene Factors

Eighteen studies evaluated the impact of various hygiene factors on levels of pesticides in environmental or biological samples, including strategies recommended in the U.S. EPA Worker Protection Standard pesticide-safety educational materials (U.S. EPA 2008) (see Supplemental Material, Table S2).

*Composite hygiene factors.* Three studies looked broadly at multiple hygiene factors. Of these three studies, the For Healthy Kids community-intervention trial of 571 farmworkers had the strongest design (Thompson et al. 2008). This 2-year educational intervention about hygiene factors had no impact on house and vehicle dust levels of three organophosphates studied (phosmet, azinphos-methyl, and malathion). Coronado et al. (2012) examined a subset of 95 homes in this population and observed that azinphos-methyl levels in house and vehicle dust were unrelated to the number of home hygiene practices undertaken (i.e., shoe removal, work clothing removal, laundering work clothes separately, vacuum and mopping frequency). In an Oregon study of 24 farm homes, McCauley et al. (2003) found no association between levels of total organophosphates or azinphos-methyl in dust and a score that incorporated work-clothes removal, shoe removal, time between arriving home and washing, and time between arriving home and changing.

*Laundering clothes.* None of the nine publications reporting the impact of laundry practices observed an association with concentrations of pesticides in residential dust (Coronado et al. 2012; Fenske et al. 2002; Lozier et al. 2012; Lu et al. 2000) or biological samples from women (Acquavella et al. 2004; Alexander et al. 2006, 2007; Semchuk et al. 2003). In CHAMACOS, women who personally laundered agricultural work clothes had 2–42% significantly higher serum levels of DDT and HCH than women who did not, but this association was not significant after adjusting for living in Mexico, where DDT had been widely used (Bradman et al. 2007).

*Changing shoes/clothes and washing after agricultural work.* Three studies observed that shoe or clothing removal was associated with pesticide concentrations in residential dust. In CHAMACOS (Harnly et al. 2009), homes of farmworkers who stored work shoes in the home had higher residential dust concentrations and loadings of chlorpyrifos, diazinon, and permethrin, but not iprodione. McCauley et al. (2003) observed that levels of azinphos-methyl and total organophosphates in residential dust were significantly lower in homes of farmworkers who changed out of their work clothes within 2 hr of arriving home from work compared with those who waited longer. No relationships with azinphos-methyl or total organophosphate levels in dust were observed in homes where workers showered within 30 min of coming home versus longer or in homes where workers reported removing shoes. Households where the farmworkers changed their work shoes inside the home had significantly higher loadings of atrazine (Lozier et al. 2012). Curwin et al. (2005) and Lozier et al. (2012) found evidence (suggestive and statistically significant) of elevated levels of pesticides in rooms where the farmer changed, compared with other rooms in the home. Five publications reported that shoe or clothing removal was unrelated to pesticide concentrations in residential dust (Coronado et al. 2012; Fenske et al. 2002; Golla et al. 2012; Lu et al. 2000) or biomarkers (Bradman et al. 2007).

*House cleaning.* Five studies provided some evidence that cleaning practices may influence levels of pesticides in residential dust. In an Oregon study, McCauley et al. (2003) observed an association between total organophosphate concentration and number of days since last cleaning of the sampled area. An Oregon cleaning intervention study in 10 homes (McCauley et al. 2006) found that cleaning windowsills significantly reduced the loadings of total organophosphates, but cleaning linoleum floors was ineffective; the effectiveness of commercial steam cleaning of the carpets was inconclusive because the baseline concentrations were low. In CHAMACOS (Harnly et al. 2009), lower cleanliness as rated by an observer considering “household organization, overflowing trash, and presence of dust” was associated with higher loadings of chlorpyrifos and dacthal but not diazinon, iprodione, or *trans*-permethrin. Quandt et al. (2004) observed that the odds of detecting a higher number of pesticides in surface wipe samples were four times higher in homes rated as difficult to clean based on age, type of dwelling, general state of repair, and crowding of occupants, furniture, and possessions; frequency of vacuuming was not associated with odds of pesticide detection. Vacuuming at least once per week was linked to reduced loadings of atrazine in residential dust in homes of pesticide handlers (not statistically significant) (Lozier et al. 2012). Five publications did not report associations between vacuuming and cleaning practices and pesticide concentrations in residential dust (Coronado et al. 2012; Curwin et al. 2005; Fenske et al. 2002; Lu et al. 2000; Simcox et al. 1995).

*Pets.* Two publications reported an association between presence of pets and concentrations of pesticides in dust. Having a dog spending time inside and outside the house was associated with two times higher atrazine levels in residential dust, compared with having no dog or a dog that stayed outside (Golla et al. 2012). Compared with having no pets, having a dog was associated with higher concentrations of chlorpyrifos and dacthal but not 11 other pesticides measured (Deziel et al. 2013). Presence of pets was not associated with concentrations of pesticides in residential dust in four studies in agricultural areas (Curwin et al. 2005; Lozier et al. 2012; McCauley et al. 2003; Simcox et al. 1995). The impact of pets may have been related to the relative time spent indoors/outdoors, which varied by study and was asked differently across the studies.

## Discussion

This literature review summarizes the evidence for the contribution of nonoccupational pathways to pesticide exposures in women living in agricultural areas, who may be exposed to a greater number of pesticides and at higher concentrations than women in the general population. A better understanding of nonoccupational pesticide exposure pathways in these women is critical to studying pesticide-related health effects and reducing exposures. Although we focused on women’s pesticide exposures, the strongest evidence came from studies with residential dust measurements, which were not specific to women. Of the 35 publications described here, 19 reported relationships between paraoccupational exposure and pesticide measurements in house dust or pesticide biomarkers; 10 observed a relationship between agricultural drift and pesticide dust concentrations; and 3 observed associations between self-reported residential use and concentrations of pesticides in dust. The relationships with hygiene factors were inconsistent across the 18 relevant studies. A large, community-intervention trial observed no impact of pesticide-safety training or hygiene factors on pesticide levels. The 4 drinking water studies generally reported poor detection rates of pesticides, providing limited information toward understanding the role of ingestion.

Evidence for the paraoccupational exposure pathway came primarily from residential dust monitoring that compared farm and non-farm homes, or homes of farmers who performed tasks that involved contact with pesticides and homes of farmers not doing those tasks. In contrast, biomonitoring studies conducted at the time of a pesticide application event did not demonstrate increases in urinary pesticide biomarkers in women whose husbands applied the chemicals compared with those who did not, even when the husbands’ exposures increased. Although these biomonitoring studies were generally well-designed, interpretation was difficult because of low percent detection and limited variability in exposures. The discrepancies between environmental and biological monitoring may be because assessment of whether the farmer-husband applied the chemical (yes/no) is not sufficiently specific to predict a concurrent increase in exposure in the spouses. For example, women who were present or outside during the pesticide application event exhibited modest increases in concentrations of pesticide biomarkers compared with women who were not present or outside (Alexander et al. 2006, 2007). Future studies should collect more detailed information about the activity and location of women when their husbands apply pesticides, as well as information on amount of pesticides applied, duration of pesticide application, hygiene factors, and use of personal protective equipment, to evaluate whether specific paraoccupational exposures increase exposure.

Agricultural drift, as measured by proximity to treated farmland, was generally associated with higher detection rates and concentrations of common agricultural pesticides in residential dust. Some studies using simple Euclidian distance did not observe an association unless additional information, such as amount of pesticides applied or acres treated, was incorporated into the exposure metrics. This is supported by results from epidemiologic studies that have demonstrated attenuation of effect estimates when proximity to fields was used as a surrogate for more refined metrics of pesticide exposure (Ritz and Rull 2008). In contrast, there was little evidence that proximity alone was linked to levels of pesticide biomarkers. Because many pesticide biomarkers reflect recent, high-exposure events (Barr and Needham 2002), associations between concentrations of pesticide biomarkers and primary agricultural drift may be expected, but not necessarily secondary drift. The relationship between biomarkers and drift are likely dependent on a variety of factors, such as timing of sample collection, application method, physicochemical properties of the pesticide, and meteorology (Ward et al. 2006). More information is needed to understand how primary and secondary components of drift contribute to residential exposure.

Moderate evidence suggested that residential pesticide use is associated with pesticide concentrations in dust in farm homes and homes in agricultural areas. Inconsistencies in relationships by pesticide may reflect *a*) whether the specific active ingredients were in the residential pest control products used, *b*) the timing of sample collection relative to when pesticides were used in the home or garden, or *c*) differences in wording of questions about residential use across studies. In addition, the dual use of several pesticides in residential and agricultural products makes it is difficult to distinguish the residential use contribution. These studies were generally small, and questions about residential use were generally nonspecific because that was not typically the study focus. More specific questions about residential pest treatments in larger study populations may improve our understanding of this relationship.

Women in agricultural areas may have different dietary patterns than the general population, for example, they may be more likely to consume fruits and vegetables directly from the field, which could contain higher pesticide residues (Goldman et al. 2004). Data related to pesticide concentrations in the diets of women living in agricultural areas were extremely limited, making conclusions difficult. We identified only one study, of six households, that provided duplicate diet measurements (Melnyk et al. 1997). Similarly, farm families often rely on private wells, which may be susceptible to pesticide contamination (Gilliom 2007). For instance, in a subset of the Agricultural Health Study cohort, 75% of participants reported using private wells as their primary source of drinking water, and 16% had wells within 50 yards of where pesticides were mixed (Gladen et al. 1998). However, the publications included in this review reported low detection rates or concentrations of pesticides in drinking water. The presence of pesticides in well water is related to many factors such as intensity of pesticide use, solubility of the pesticide, and permeability of the soil (Stackelberg et al. 2012). More studies on food and drinking water–based exposures in agricultural populations would help inform the role of the ingestion pathway.

Although hygiene factors are a potential exposure pathway modifier, our review identified limited support for relationships between pesticide levels in dust and shoe/clothing removal, laundry practices, and presence of pets. Five studies did suggest that house cleaning practices may be related to pesticides in the dust. However, many studies were not focused on hygiene factors, had limited power to evaluate these practices, and incorporated questions that were subjective or were asked differently across the studies. One exception was the For Healthy Kids study (Coronado et al. 2012; Thompson et al. 2008), a relatively large, community-based intervention study specifically evaluating whether safety and hygiene factors were associated with pesticide exposures. This study found that neither recommended practices, such as removing shoes and laundering work clothes separately, nor an educational intervention were linked to pesticide levels in homes or commuter vehicles. These findings suggest that more work is needed to investigate the effectiveness of recommended practices.

Some challenges warrant consideration in interpretation of this review. Disentangling the exposure pathways remains difficult because agricultural populations are concurrently exposed to pesticides via multiple pathways. In addition, pesticide levels in both residential dust and biomarkers aggregate over multiple pathways; therefore, independent contributions from each pathway are not easy to discern. We observed some inconsistent relationships between environmental and biological measurements, which may reflect different windows of exposure. Dust may capture the accumulation of many sources over time (Simcox et al. 1995), and biomarkers for most current-use pesticides reflect recent exposures due to their relatively short half-lives (Barr and Needham 2002). Agreement may be expected only if daily exposures were fairly stable within an individual. In addition, pesticide dust levels, although a potentially useful exposure indicator in children (Bradman et al. 1997), may not be a good proxy for exposure in adults. People may be exposed to pesticides in dust via incidental ingestion, dermal absorption, and inhalation, but the extent of these exposures in adults and their dependence on individual specific activity factors is not well understood (U.S. EPA 2011). Although both dust and biomarkers aggregate over multiple pathways, only biomarkers are aggregated via dietary or occupational pathways; thus, differences may occur when either of these pathways contribute substantially to total exposure. In addition, few studies had dust and biological samples in the same population; thus, inconsistencies could be attributable to any of the many factors that differed among the studies (e.g., geographical location, study time period, diet). Curwin et al. (2007) measured both dust and biomarkers and found that, for farm women, urinary levels were not associated with house dust levels of any pesticides. In non-farm women, the pesticide urinary levels of metolachlor were associated with dust levels, but those of atrazine, chlorpyrifos, and glyphosate were not. Ultimately, dust and biomarker measurements may provide complementary information. Methodological studies to better understand the relationship between these two metrics are needed to interpret this body of literature.

Studies that attempted to isolate a pathway through stratification or adjustment in multivariable models and/or studies with both biological and environmental measurements, such as CHAMACOS, For Healthy Kids, the University of Washington studies, and the Iowa farm family exposure study, provided the most information on the relative importance of pathways. Apparent inconsistencies across studies that used the same exposure measures may be due to differences in study population and sample size; pesticides measured; regional or temporal differences in pesticide-use patterns; differences in sampling, laboratory, and statistical methods; differences in the way the nonoccupational pathways were assessed; product formulations; or the physicochemical properties of the pesticides. For example, although farmers and farmworkers could perform different tasks, leading to different paraoccupational exposures, our review combined these two occupational groups due to lack of standardization in definitions in the literature. There was insufficient evidence in the literature to examine pathways for individual pesticides.

Other gaps in understanding warrant consideration. Most studies measured only a few pesticides, and many active ingredients currently in common use are not covered by the literature reviewed here. The studied populations were concentrated in certain geographical areas (e.g., northwestern United States) with distinct crop types; therefore, this body of literature may not be generalizable to all agricultural areas in North America or to other parts of the world. In addition, this review did not focus on the occupational pathway, but because women living on farms may personally handle pesticides, there is a need in future research to place the nonoccupational exposure pathways into context with the occupational pathway. Finally, publication bias could be a potential source of error in this review if the published research we surveyed is not representative of all completed studies.

## Conclusion

Pesticides have been linked to numerous adverse health effects, and effects could be different in women than in men. Although the potential for relatively high exposure to pesticides in agricultural women compared with the general population has been documented, exposure characterization has been limited. Therefore, we undertook an extensive review to better understand the contribution of nonoccupational pathways to pesticide exposure in women living in agricultural areas. Relative to the body of literature on male farmers and farm children, women living in farm homes or in agricultural areas remain largely understudied. Most of the evidence reviewed came from studies of residential dust, which is not specific to women. The results from biomonitoring studies specifically of women were often difficult to interpret because of low detection rates or limited variability in pesticide biomarkers. Future research should include women with a greater variability in pesticide contact and should include more detailed information about the extent of their contact with pesticides, either on the farm or in the home. Although disentangling exposure pathways was challenging, overall, we found reasonably consistent evidence that paraoccupational and agricultural drift pathways contributed to pesticide exposure in women, with moderate consistency for the contribution of residential pesticide use and limited evidence for hygiene factors as an exposure modifier. Insufficient evidence was available to assess dietary exposures. Our literature review identified sufficient papers to empirically derive weights for nonoccupational exposure pathways.

An improved understanding of the important pathways of pesticide exposure in women is critical for future epidemiologic and exposure studies as well as for designing effective risk mitigation strategies.

## Supplemental Material

(292 KB) PDF
