# Supplementary material for: A Review of Nonoccupational Pathways for Pesticide Exposure in Women Living in Agricultural Areas
Source: Environ Health Perspect. 2015 Jan 30;123(6):515–24. doi: 10.1289/ehp.1408273 (PMC4455586; doi:10.1289/ehp.1408273)
Supplement: (292 KB) PDF [file ehp.1408273.s001.508.pdf]

Environmental Health Perspectives doi:10.1289/ehp.1408273

Supplemental Material

A Review of Nonoccupational Pathways for Pesticide Exposure in Women Living in Agricultural Areas

Nicole C. Deziel, Melissa C. Friesen, Jane A. Hoppin, Cynthia J. Hines, Kent Thomas, and Laura E. Beane Freeman

Table S1. Pesticides evaluated in each reviewed publication.

Study, Source	Herbicides	Insecticides	Fungicides
**Agricultural Health Pilot Study**			
Melnyk et al. 1997	2,4-D, alachlor, atrazine, dacthal, dicamba, metolachlor, trifluralin	a-chlordane, aldicarb, aldrin, carbaryl, carbofuran, chlorpyrifos, DDD, DDE, DDT, dieldrin, fonofos, g-chlordane, heptachlor, lindane, malathion, permethrin, phorate, propoxur, pyrethrins, terbufos	captan, chlorothalonil, dicloran, folpet, metalaxyl
**California Childhood Leukemia Study**			
Gunier et al. 2011	dacthal, simazine	carbaryl, chlorpyrifos, diazinon, phosmet	iprodione
**CHAMACOS**			
Bradman et al. 2007	NA	DDE, DDT, HCB, HCH	NA
Harnly et al. 2009	dacthal	acephate, azinphos-methyl, bensulide, chlorpyrifos, cis-permethrin, DCPA, DDE, DDT, diazinon, dimethoate, fenamiphos, fonofos, iprodione, malathion, methamidiphos, methidathion, methomyl, oxydemeton-methyl, phosmet, trans-permethrin	iprodione, vinclozoline
Huen et al. 2012	NA	chlorpyrifos, diazinon	NA
**Farm Family Exposure Study**			
Acquavella et al. 2004	glyphosate	NA	NA
Alexander et al. 2006	NA	chlorpyrifos	NA
Alexander et al. 2007	2,4-D	NA	NA
**For Healthy Kids Study**			
Coronado et al. 2006	NA	azinphos-methyl (low detection rates: chlorpyrifos, diazinon, malathion, methyl parathion, phosmet)	NA
Coronado et al. 2011	NA	azinphos-methyl (low detection rates: DMPT, phosmet)	NA
Coronado et al. 2004	NA	azinphos-methyl (low detection rates: chlorpyrifos, diazinon, malthion, methyl parathion, phosmet)	NA

Study, Source	Herbicides	Insecticides	Fungicides
Curl et al. 2002	NA	azinphos-methyl, chlorpyrifos, diazinon, malathion, methyl parathion, phosmet	NA
Thompson et al. 2008	NA	azinphos-methyl, malathion, phosmet	NA
Coronado et al. 2012	NA	azinphos-methyl	NA
**Fresno Pesticide Exposure Study**			
Deziel et al. 2013	dacthal, simazine, trifluralin	carbaryl, chlordane, chlorpyrifos, cyfluthrin, cypermethrin, diazinon, methoxychlor, permethrin, piperonyl butoxide, propoxur	NA
**Iowa Farm Family Pesticide Exposure Study**			
Curwin et al. 2007	2,4-D, acetochlor, alachlor, atrazine, glyphosate, metolachlor	chlorpyrifos	NA
Curwin et al. 2005	2,4-D, acetochlor, alachlor, atrazine, glyphosate, metolachlor	chlorpyrifos	NA
**Iowa Pesticide Exposure Studies**			
Lozier et al. 2012	atrazine	NA	NA
Golla et al. 2012	atrazine	NA	NA
**Non-Hodgkin Lymphoma Study**			
Ward et al. 2006	2,4-D, acetochlor, alachlor, atrazine, bentazon, dicamba, fluazifop-p-butyl, metolachlor, pendimethalin, trifluralin	NA	NA
**Ontario Pesticide Exposure Assessment Study**			
Arbuckle et al. 2006	2,4-D	NA	NA
Arbuckle and Ritter 2005	2,4-D, MCPA	NA	NA
**Oregon Exposure Studies**			
McCauley et al. 2001	NA	azinphos-methyl (low detection rates: captan, carbaryl, chlorpyrifos, DDE, DDT, malathion, pentachlorophenol, phosmet, pipernoyl butoxide)	NA
McCauley et al. 2003	NA	azinphos-methyl, chlorpyrifos, diazinon, malathion, parathion, phosmet	NA
McCauley et al. 2006	NA	azinphos-methyl, chlorpyrifos, diazinon, ethyl parathion, malathion, methyl parathion, phosmet	NA
**University of Washington Studies**			
Fenske et al. 2002	NA	chlorpyrifos, ethyl parathion	NA

Study, Source	Herbicides	Insecticides	Fungicides
Lu et al. 2000	NA	azinphos-methyl, phosmet	NA
Lu et al. 2004	NA	azinphos-methyl, chlorpyrifos, diazinon, phosmet	NA
Simcox et al. 1995	NA	azinphos-methyl, phosmet, chlorpyrifos, ethyl parathion	NA
Weppner et al. 2006	NA	methamidophos	NA
**Other Studies**			
Freeman et al. 2004	atrazine, simazine	azinphos-methyl, chlorpyrifos, demeton-O, ethion, demeton-S, diazinon, disulfoton, ethyl parathion, fenithrothion, fonofos, malathion, methyl parathion	NA
Quandt et al. 2004	atrazine, metolachlor, oxyfluorfen, pendimethalin, simazine	a-chlordane, carbaryl, chlorpyrifos, cis-permethrin, DDE, DDT, diazinon, esfenvalerate, g-chlordane, heptachlor, lindane, methoxychlor, propoxur, total disulfoton, trans-permethrin	ortho-phenylphenol
Richards et al. 2001	propanil	NA	NA
Semchuk et al. 2003	2,4-D, bromoxynil, dicamba, ethalfluralin, fenoxaprop, triallate, trifluralin, MCPA	NA	NA
Fitzgerald et al. 2001	2,4-D, bromoxynil, dicamba, diclofop-methyl, fenoxyprop, MCPA, triallate, trifluralin	NA	NA

2,4-D, 2,4-dichlorophenoxyacetic acid; DCPA, 2,3,5,6-tetrachloroterephthalate; DDE, dichlorodiphenyldichloroethylene; DDT, dichloro-diphenyl-trichloroethane; DMPT, dimethylphosphorothidate, DDD, dichlorodiphenyldichloroethane; HCB, hexachlorobenzene, HCH, hexachlorocyclohexane; MCPA, 2-methyl-4-chlorophenoxyacetic acid; NA, not applicable

Table S2. Evidence for the relationship between pesticide levels in biological or environmental samples and hygiene factors in the reviewed literature.[a]

Study, Source	Overall	Laundry	Changing Clothes/ Shoes	House Cleaning	Pets
**CHAMACOS**					
Bradman et al. 2007	NA	o	o	NA	NA
Harnly et al. 2009	NA	NA	+	+	NA
**Farm Family Exposure Study**					
Acquavella et al. 2004	NA	o	NA	NA	NA
Alexander et al. 2006	NA	o	NA	NA	NA
Alexander et al. 2007	NA	o	NA	NA	NA
**For Healthy Kids Study**					
Coronado et al. 2012	o	o	o	o	NA
Thompson et al. 2008	o	NA	NA	NA	NA
**Fresno Pesticide Exposure Study**					
Deziel et al. 2013	NA	NA	NA	NA	+
**Iowa Pesticide Exposure Studies**					
Lozier et al. 2012	NA	o	+	+	o
Golla et al. 2012	NA	NA	o	NA	+
**Iowa Farm Family Pesticide Exposure Study**					
Curwin et al. 2005	NA	NA	descriptive	o	o
**Oregon Exposure Studies**					
McCauley et al. 2003	o	NA	+	+	o
McCauley et al. 2006	NA	NA	NA	+	NA
**University of Washington Exposure Studies**					
Fenske et al. 2002	NA	o	o	o	NA
Lu et al. 2000	NA	o	o	o	NA
Simcox et al. 1995	NA	NA	NA	o	o
**Other Studies**					
Quandt et al. 2004	NA	NA	NA	+	
Semchuk et al. 2003	NA	o	NA	NA	NA

[a]Symbol "+" indicates association between pesticide levels and the exposure pathway was observed for at least one pesticide (p<0.1); "o", no associations between pesticide levels and exposure pathway observed (p>0.1).
CHAMACOS, Center for the Health Assessment of Mothers and Children of Salinas; NA, not applicable.

References

Acquavella JF, Alexander BH, Mandel JS, Gustin C, Baker B, Chapman P, et al. 2004. Glyphosate biomonitoring for farmers and their families: results from the Farm Family Exposure Study. Environ Health Perspect. 1123:321-326.

Alexander BH, Burns CJ, Bartels MJ, Acquavella JF, Mandel JS, Gustin C. et al. 2006. Chlorpyrifos exposure in farm families: results from the farm family exposure study. J Expo Sci Environ Epidemiol. 165:447-456.

Alexander BH, Mandel JS, Baker BA, Burns CJ, Bartels MJ, Acquavella JF, et al. 2007. Biomonitoring of 24-dichlorophenoxyacetic acid exposure and dose in farm families. Environ Health Perspect. 1153:370-376.

Arbuckle TE, Bruce D, Ritter L, Hall JC. 2006. Indirect sources of herbicide exposure for families on Ontario farms. J Expo Sci Environ Epidemiol 161:98-104.

Arbuckle TE and Ritter L. 2005. Phenoxyacetic acid herbicide exposure for women on Ontario farms. J Toxicol Environ Health A 6815:1359-1370.

Bradman AS, Schwartz JM, Fenster L, Barr DB, Holland NT, Eskenazi B. 2007. Factors predicting organochlorine pesticide levels in pregnant Latina women living in a United States agricultural area. J Expo Sci Environ Epidemiol. 174:388-399.

Coronado GD, Holte S, Vigoren E, Griffith WC, Barr DB, Faustman E et al. 2011. Organophosphate pesticide exposure and residential proximity to nearby fields: evidence for the drift pathway. J Occup Environ Med 538:884-891.

Coronado GD, Holte SE, Vigoren EM, Griffith WC, Barr DB, Faustman EM, et al. 2012. Do workplace and home protective practices protect farm workers? Findings from the "For Healthy Kids" study. J Occup Environ Med 549:1163-1169.

Coronado GD, Thompson B, Strong L, Griffith WC, Islas I. 2004. Agricultural task and exposure to organophosphate pesticides among farmworkers. Environ Health Perspect 1122:142-147.

Coronado GD, Vigoren EM, Thompson B, Griffith WC, Faustman EM. 2006. Organophosphate pesticide exposure and work in pome fruit: evidence for the take-home pesticide pathway. Environ Health Perspect 1147:999-1006.

Curl CL, Fenske RA, Kissel JC, Shirai JH, Moate TF, Griffith W, et al. 2002. Evaluation of take-home organophosphorus pesticide exposure among agricultural workers and their children. Environ Health Perspect. 11012 A787-A792.

Curwin BD, Hein MJ, Sanderson WT, Nishioka MG, Reynolds SJ, Ward EM, et al. 2005. Pesticide contamination inside farm and nonfarm homes. J Occup Environ Hyg 27:357-367.

Curwin BD, Hein MJ, Sanderson WT, Striley C, Heederik D, Kromhout H et al. 2007. Urinary pesticide concentrations among children mothers and fathers living in farm and non-farm households in iowa. Ann Occup Hyg 511:53-65.

Deziel NC, Ward MH, Bell EM, Whitehead TP, Gunier RB, Friesen MC, et al. 2013. Temporal variability of pesticide concentrations in homes and implications for attenuation bias in epidemiologic studies. Environ Health Perspect 121:565-571

Fenske RA, Lu C, Barr D, Needham L. 2002. Children's exposure to chlorpyrifos and parathion in an agricultural community in central Washington State. Environ Health Perspect 1105:549-553.

Fitzgerald D, Chanasyk DS, Neilson RD, Kiely D, Audette R. 2001. Farm well water quality in Alberta. Water Quality Research Journal of Canada 363:565-588.

Freeman NC, Shalat SL, Black K, Jimenez M, Donnelly KC, Calvin A et al. 2004. Seasonal pesticide use in a rural community on the US/Mexico border. J Expo Anal Environ Epidemiol 146:473-478.

Golla V, Curwin B, Nishioka M. 2012. Pesticide concentrations in vacuum dust from farm homes: variation between planting and nonplanting Seasons. ISRN Public Health 2012: 539397.

Gunier RB, Ward MH, Airola M, Bell EM, Colt J, Nishioka M et al. 2011. Determinants of agricultural pesticide concentrations in carpet dust. Environ Health Perspect 1197:970-976.

Harnly ME, Bradman A, Nishioka M, McKone TE, Smith D, McLaughlin R. et al. 2009. Pesticides in dust from homes in an agricultural area. Environ Sci Technol 4323:8767-8774.

Huen K, Bradman A, Harley K, Yousefi P, Barr DB, Eskenazi B et al. 2012. Organophosphate pesticide levels in blood and urine of women and newborns living in an agricultural community. Environ Res 117:8-16.

Lozier MJ, Curwin B, Nishioka MG, Sanderson W. 2012. Determinants of atrazine contamination in the homes of commercial pesticide applicators across time. J Occup Environ Hyg 95:289-297.

Lu C, Fenske RA, Simcox NJ, Kalman D. 2000. Pesticide exposure of children in an agricultural community: evidence of household proximity to farmland and take home exposure pathways. Environ Res. 843:290-302.

Lu C, Kedan G, Fisker-Andersen J, Kissel JC, Fenske RA. 2004. Multipathway organophosphorus pesticide exposures of preschool children living in agricultural and nonagricultural communities. Environ Res. 963:283-289.

McCauley LA, Lasarev MR, Higgins G, Rothlein J, Muniz J, Ebbert C et al. 2001. Work characteristics and pesticide exposures among migrant agricultural families: a community-based research approach. Environ Health Perspect 1095:533-538.

McCauley LA, Michaels S, Rothlein J, Muniz J, Lasarev M, Ebbert C. 2003. Pesticide exposure and self reported home hygiene: practices in agricultural families. AAOHN J. 513:113-119.

McCauley LA, Travers R, Lasarev M, Muniz J, Nailon R. 2006. Effectiveness of cleaning practices in removing pesticides from home environments. J Agromedicine: 112 81-88.

Melnyk LJ, Berry MR, Sheldon LS. 1997. Dietary exposure from pesticide application on farms in the Agricultural Health Pilot Study. J Expo Anal Environ Epidemiol. 71:61-80.

Quandt SA, Arcury TA, Rao P, Snively BM, Camann DE, Doran AM et al. 2004. Agricultural and residential pesticides in wipe samples from farmworker family residences in North Carolina and Virginia. Environ Health Perspect. 1123: 382-387.

Richards SM, McClure GY, Lavy TL, Mattice JD, Keller RJ Gandy J. 2001. Propanil 34-dichloropropionanilide particulate concentrations within and near the residences of families living adjacent to aerially sprayed rice fields. Arch Environ Contam Toxicol 411:112-116.

Semchuk KM, McDuffie HH, Senthilselvan A, Dosman JA, Cessna AJ, and Irvine DG. 2003. Factors associated with detection of bromoxynil in a sample of rural residents. J Toxicol Environ Health A. 662: 103-132.

Simcox NJ, Fenske RA, Wolz SA, Lee IC, Kalman DA. 1995. Pesticides in household dust and soil: exposure pathways for children of agricultural families. Environ Health Perspect 10312:1126-1134.

Thompson B, Coronado GD, Vigoren EM, Griffith WC, Fenske RA, Kissel JC, et al. 2008. Para ninos saludables: a community intervention trial to reduce organophosphate pesticide exposure in children of farmworkers. Environ Health Perspect. 1165:687-694.

Ward MH, Lubin J, Giglierano J, Colt JS, Wolter C, Bekiroglu N et al. 2006. Proximity to crops and residential exposure to agricultural herbicides in Iowa. Environ Health Perspect. 1146:893-897.

Weppner S, Elgethun K, Lu C, Hebert V, Yost MG, Fenske RA. 2006. The Washington aerial spray drift study: children's exposure to methamidophos in an agricultural community following fixed-wing aircraft applications. J Expo Sci Environ Epidemiol 165:387-396.
